# Outcome in recurrent head neck cancer treated with salvage-IMRT

**DOI:** 10.1186/1748-717X-3-43

**Published:** 2008-12-17

**Authors:** Gabriela Studer, Klaus W Graetz, Christoph Glanzmann

**Affiliations:** 1 Department of Radiation Oncology, University Hospital Zurich, Zurich, Switzerland; 2 Department of Craniomaxillofacial Surgery, University Hospital, Zurich, Switzerland

## Abstract

**Background:**

Recurrent head neck cancer (rHNC) is a known unfavourable prognostic condition.

The purpose of this work was to analyse our rHNC subgroup treated with salvage-intensity modulated radiation therapy (IMRT) for curable recurrence after initial surgery alone.

**Patients:**

Between 4/2003–9/2008, 44 patients with squamous cell rHNC were referred for IMRT, mean/median 33/21 (3–144) months after initial surgery. None had prior head neck radiation. 41% underwent definitive, 59% postoperative IMRT (66–72.6 Gy). 70% had simultaneous chemotherapy.

**Methods:**

Retrospective analysis of the outcome following salvage IMRT in rHNC patients was performed.

**Results:**

After mean/median 25/21 months (3–67), 22/44 (50%) patients were alive with no disease; 4 (9%) were alive with disease. 18 patients (41%) died of disease. Kaplan Meier 2-year disease specific survival (DSS), disease free survival (DFS), local and nodal control rates of the cohort were 59/49/56 and 68%, respectively.

Known risk factors (advanced initial pTN, marginal initial resection, multiple recurrences) showed no significant outcome differences. Risk factors and the presence of macroscopic recurrence gross tumor volume (rGTV) in oral cavity patients vs others resulted in statistically significantly lower DSS (30 vs 70% at 2 years, p = 0.03). With respect to the assessed unfavourable outcome following salvage treatment, numbers needed to treat to avoid one recurrence with initial postoperative IMRT have, in addition, been calculated.

**Conclusion:**

A low salvage rate of only ~50% at 2 years was found. Calculated numbers of patients needed to treat with postoperative radiation after initial surgery, in order to avoid recurrence and tumor-specific death, suggest a rather generous use of adjuvant irradiation, usually with simultaneous chemotherapy.

## Background

In deciding on postoperative irradiation in patients with head neck cancer (HNC), the risk of recurrence as well as the results of treatment of a recurrence are the most important criteria. Local recurrence seems to have an unfavourable prognosis: there is general accordance in the literature, that success rates of salvage treatment of recurrent HNC (rHNC) are low. More than 50% of rHNC patients who undergo salvage treatment, will die after salvage treatment as a direct consequence of local-regionally recurrent disease [1-8]. There are, however, divergent data with respect to the factors, that may influence the prognosis of rHNC following salvage therapy, like initial TN stages, recurrence TN stages, primary site, recurrence salvage treatment, time to recurrence, site of recurrence, or resection margins, respectively [1-6,8-10].

The comparability of reported results in the literature is limited, as examined collectives substantially differ with respect to initial TN stages, initial as well as recurrence treatment strategies, or primary site, respectively. Furthermore, the sample size of most reported collectives is small.

Recent publications report a marked improvement of local-regional control after intensity modulated radiation therapy (IMRT) in the initial treatment of HNC [11-18].

We analysed the outcome of our definitively or postoperatively treated IMRT subgroup referred for local or regional recurrence after initial surgery alone, in order to evaluate the salvage rate following modern treatment methods.

## Patients

Between 4/2003 and 9/2008, 44 patients with rHNC were referred for IMRT to the Department of Radiation Oncology, University Hospital Zurich, mean/median 33/21 (3–144) months after initial surgical treatment. The rHNC subgroup represents 8% (44/530) of all IMRT patients who were referred for a curatively intended irradiation of a squamous cell HNC during the indicated time period. None of the rHNC patients had prior radiation therapy of the HN region.

Mean age at diagnosis of rHNC was 64.3 years (35–87). The male to female ratio was ~2:1 (28 :16). Tumor related parameters are listed in Table 1a and 1b.

**Table 1:** 

**a **Tumor related parameters					
**Parameters**	**n (%)**				

**Diagnosis**					
oral cavity	29 (66)				
glottic	8 (18)				
lateral oropharynx	4 (9)				
sinonasal	2 (5)				
skin	1 (2)				
					
**initial resection**					
wide (>2 mm, RO)	13				
marginal (R1)	16				
intralesional (R2)	0				
unknown	15				
					
**initial pT**					
pT1	14				
pT2	23				
pT3	1				
pT4	3				
unknown	3				
					
**initial p/cN**					
N0	26				
N1	5				
N2a/b	9				
N2c	2				
unknown	2				
					
**initial grading**					
G1	6				
G2	15				
G3	12				
unknown	11				
					
**grading recurrence**					
G1	2				
G2	21				
G3	11				
unknown	10				
					
**No. recurrence**					
1st	31				
2nd	8				
3rd	4				
4th	1				
					
**site of recurrence**					
nodal	14				
mucosal	16				
nodal and mucosal	14				

Tumor characteristics in 44 patients referred for recurred squamous cell carcinoma of the head neck (rHNC).					

**b **Tumor related parameters					

**rTN**	**rN0**	**rN1**	**rN2ab**	**N2c**	**Total**

**rT0**	0	4	5	5	**14**
**rT1**	1	0	0	1	**2**
**rT2**	1	0	1	1	**3**
**rT3**	0	1	0	0	**1**
**rT4**	18	2	3	0	**23**
**rT?**	0	0	1	0	**1**

**Total**	**20**	**7**	**10**	**7**	**44**

Recurrence stages (rTN) of the 44 assessed patients. 23/44 (52%) presented with rT4 stage.					

In order to retrospectively assess parameters that could help to predict outcome following salvage therapy, we assessed generally accepted prognostic parameters like early vs more advanced TN stages, histo-pathological grading, the number of prior recurrences, resection status (close vs wide margins), diagnosis, and, in addition, the location of recurrence (nodal vs primary), respectively.

## Methods

Several disease related factors were analysed with respect to their prognostic impact on outcome following salvage therapy. Considering the small sample size (n = 44), single risk factors were grouped to 'high risk' vs 'low risk' features (Table 2).

**Table 2:** Patients grouped according to risk parameters

**Parameters**	**low risk (n)**	**high risk (n)**
**initial pTN**	</=pT2N0 or T1N0-2b (14)	>pT2N0 (14)
**initial resection**	and R0 (6) or unknown (8)	and/or R1 (16)
**initial grading (G)**	and G1-2 (7) or unknown (6)	and any G
**No. of recurrence**	and all 1st (14)	any 1st (17) or (2nd–4th (13)

**patients (n)**	**32% (14)**	**68% (30)**

Grouped 'low' vs 'high' risk parameters in the assessed cohort.

### Salvage treatment of rHNC

In 18/44 (41%) rHNC, definitive salvage IMRT was performed, 26 (59%) patients underwent IMRT following salvage surgery of recurrent primary and/or nodal disease. All 26 postoperative IMRT patients underwent macroscopically complete salvage surgery (R1-R0), however, in 12/26 patients, the planning computed tomography (planning-CT) showed already re-grown nodal or local recurrence gross tumor volume (rGTV). Re-staging and re-resection in all but 2 patients have been performed by surgical experts of the associated clinics of maxillofacial or head neck surgery at the University Hospital Zurich. Re-staging was based on clinical examination, histopathological confirmation of the lesions, and computed tomography and/or magnetic resonance imaging and/or positron emission tomography in all patients. The mean interval between salvage surgery and postoperative salvage IMRT was 5 weeks (3–8).

IMRT was performed according to our institutional standard schedules, that are routinely used for IMRT of initial HNC disease in curative intention: for postoperative IMRT (+/- systemic therapy), this is 66 Gy in 33 fractions to the boost volume (5×/week), for definitive IMRT (+/- systemic therapy), schedules with 33× 2.11-2.2 Gy (5×/week), or 35× 2.0 Gy (5–6×/week), respectively, are used. Postoperative patients with re-grown rGTV detectable in the planning computed tomography were treated like the 'definitive IMRT' subgroup (i.e. with tumor doses up to 70–72.6 Gy).

All schedules are based on simultaneously integrated boost (SIB) delivery [15]. In ~70%, simultaneous systemic therapy was given (in 27/44 cisplatin (40 mg/m2/w), in 3/44 erbitux (3–6 cycles: 1×400 mg/m2 and 2–5×250 mg/m2/w; indications: contraindications against cisplatin, intolerance of cisplatin).

### Statistics

Actuarial survival data were calculated using Kaplan-Meier curves implemented in StatView^® ^(Version 4.5). p values < 0.05 were considered statistically significant.

## Results

**Figure 1:**
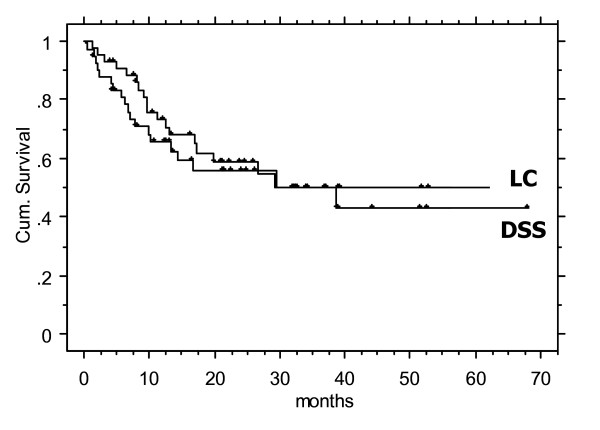
Local control (LC) and disease specific survival (DSS) in 44 patients treated with IMRT for recurred HNC (rHNC).

After mean/median 25/21 months (3–67) following salvage IMRT, 22/44 patients (50%) were alive with no evidence of disease when last seen; 4 (9%) were alive with disease, respectively. 18 patients (41%) died from disease mean 9.8 months (1.3–29) after salvage treatment. Disease specific survival (DSS), disease free survival (DFS), local and nodal control rates of the entire cohort following IMRT were 59/49/56 and 68% at 2 years, respectively (calculated using Kaplan-Meier survival curves, Figure 1).

Known unfavourable factors per se, like advanced initial pTN stages (n = 13), initial marginal resection (R1, n = 16), or >1 recurrence prior to salvage radiation (n = 13), did not result in statistically significant outcome differences. The combination of these factors ('high risk ', Table 2), as well as presence of visible recurrence gross tumor volume (rGTV) in the planning CT (n = 30), were tendentially unfavourable predictors for DSS or DFS (p ~0.1 each).

Significant statistical 2-year local control (LC) rates and DSS differences were found for 'high risk profile' OCC patients (risks as listed in Table 2) with measurable rGTV (n = 14) vs others (n = 30; 30 vs 60% (p = 0.05), and 30 vs 70%, p = 0.03).

**Figure 2:**
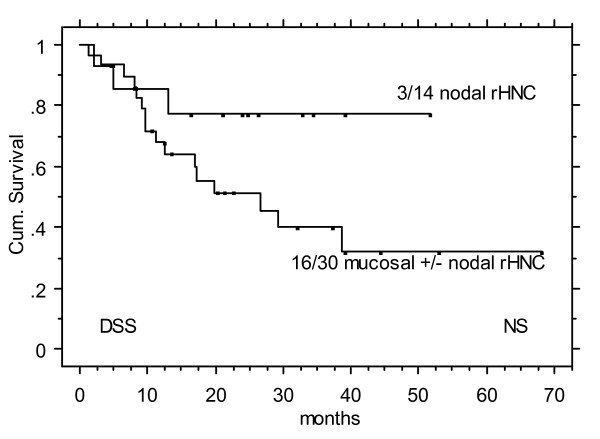
Disease specific survival (DSS) following salvage IMRT for isolated nodal recurrence vs mocosal (i.e. primary tumor) +/- nodal recurrence.

The site of recurrence (nodal (n = 14) vs mucosal (i.e. primary site) +/- nodal (n = 30)) showed a non- significant DSS difference in favour of patients with nodal recurrence only (~80 vs ~40% at 2 years, Figure 2).

Kaplan Meyer 2-year DFS/DSS rates did not differ for rT0-2rN0-2b vs rT3-4rN0-2c or rT0-2 vs rT3-4, or rN stages, or primary vs postoperative salvage IMRT, respectively.

The potential impact of concomitant systemic therapy could not be evaluated based on this small series with different local treatment approaches (Table 3).

**Table 3:** Patients listed according to the performed different treatment modalities

**Treatment modality**	**n**	**failures after salvage treatment (22/44)**
**Biopsy only + IMRT**	5	3 of 5
**Biopsy only + IMRT + Cisplatin (10) or Erbitux (3)**	13	8 of 13
		
**R0-1 resection + IMRT**	3	0 of 3
**R0-1 resection + IMRT + Cisplatin**	11	5 of 11
		
**R0-1 resection with GTV in Pl-CT + IMRT**	4	3 of 4
**R0-1 resection with GTV in Pl-CT + IMRT + Cisplatin**	8	3 of 8

All 44 rHNC patients, analysed according to the performed salvage treatment modalities. The numbers per treatment modality arm are too small to draw reliable conclusions with respect to the impact of concomitant chemotherapy.

Pl-CT: Planning-computed tomography

## Discussion

### - Outcome following salvage treatment

The presented cohort was fairly homogeneous with respect to the previous surgical treatment and performed IMRT with 66–72.6 Gy, respectively. As expected, at the time of primary surgery, the majority of patients presented with an early stage of cancer (32/44 </= pT2N2a (73%), 5 unknown).

Limitations of the study are the small sample size and short follow up; however, the number of re-recurrent events was high with 18 local, 14 nodal, and 4 distant failures (36 events in 22 patients), that mostly occurred during the first year post salvage therapy (33/36, 92%).

2-year DSS and local control following salvage treatment in the entire collective were as low as ~50% (Figure 1), comparable to the outcome in our IMRT patients treated with primary radio-chemotherapy for a very large initial primary GTV of >70cc [19]. The presented outcome after definitive as well as postoperative salvage IMRT confirms reported general re-recurrence rates after salvage radiation therapy in non-IMRT cohorts [1-8].

Inferior salvage outcome in recurred OCC primaries is reported from other centres [2,4]. The combination of initial 'high risk' profile plus measurable rGTV in OCC revealed to be a particularly unfavourable prognostic constellation with respect to DSS, DFS and local control in our cohort; the collective is too small yet to perform analyses according to the different diagnoses, although there is already a tendency to inferior outcome for OCC – in accordance to the formerly reported inferior outcome of OCC vs other entities in the initial treatment setting [20]. Non-OCC patients with widely resected early stage initial disease, that underwent macroscopically radical salvage surgery and postoperative IMRT for a first recurrence (n = 14), represented a favourable rHNC subgroup with ~70% DSS at 2 years.

Definitive salvage radiation has been reported less effective than salvage surgery +/- postoperative radiation by several other authors [2,3,8,21]. Our results showed no significant difference, likely due to the small samples with even re-grown gross tumor volumes in 12 of 26 operated patients.

The tendency to an outcome difference between nodal vs mucosal (i.e. primary) +/- nodal recurrence (Figure 2, p = 0.03) may confirm results by Regine et al [1]. These authors analysed 31 rHNC patients with surgically treated initial lesions, and found significant differences in local control (p = 0.001) and DSS (p = 0.0001) in favour to nodal only recurrences (n = 13).

### - Numbers needed to treat to avoid recurrence

With respect to the assessed unfavourable outcome of rHNC following postoperative or definitive salvage IMRT, avoidance of recurrence becomes more meaningful. In order to estimate the number needed to treat (NNT) to avoid one recurrence with postoperative IMRT in the initial situation, NNTs have been approximatively calculated from the here presented data (Table 4). Calculations based on the presented recurrence rate of 50% following salvage treatment (row 'cure rate' in the table). For initial situations with estimated ~10% loco-regional failure rate, an about 10% distant spread probability was provided, for situations with a higher loco-regional failure rate of ~30%, a higher rate of ~20% distant spread was estimated ('100-10' vs '100-20 patients', first row in the table). The estimated loco-regional recurrence rate bases on own data on our postoperatively IMRT-treated patient cohort [16]. For favourable initial pT1-2N0M0 stage patients, recurrence rates of ~10–20% and more are reported in recent surgical literature [21-24]. Provided an accepted recurrence rate of ~10%, most early stage HNC patients should, in consequence, undergo initial postoperative radiation. The calculated NNT are, – considering also the improved treatment tolerance following IMRT [15,25-28]-, suggestive for initial postoperative IMRT in most early stage HNC (except of glottic tumors), especially in OCC primaries.

**Table 4:** Numbers needed to treat (NNT)

**N patients**	**treatment**	**estimated loco-reg RR **(examples)	**n R**	**n treated R**	**cure rate R **(n)	**survival **(n)	**NNT**
**100**	OP only	10%	10	10	50% (5)	95% (95)	**21**
**100**	postop RT	5%	0.5	0.5	50% (0.25)	99.75% (99.75)	
**100 – 10 M+**	OP only	10%	10	10	50% (5)	85% (85)	**21**
**100 – 10 M+**	postop RT	5%	0.5	0.5	50% (0.25)	89.75% (89.75)	
**100**	OP only	30%	30	30	50% (15)	85% (85)	**8**
**100**	postop RT	15%	5	5	50% (2.5)	97.5% (97.5)	
**100 – 20 M+**	OP only	30%	30	30	50% (15)	65% (65)	**8**
**100 – 20 M+**	postop RT	15%	5	5	50% (2.5)	77.5% (77.5)	

Estimated numbers needed to treat (NNT), calculated for two populations with an estimated risk for recurrence (RR) of 10% (white) or 30% (grey), respectively (RR of 5% in postoperative IMRT cohorts has been derived from the own postoperative IMRT pT1-2 -fraction [16]). The calculations were performed each with and without considering the distant metastasis (M+) fraction; the percentage of M+ was based on the observed M+ rate in the own IMRT population; with higher local-regional risk (e.g. 30%), the M+ rate is usually also expected increasing (e.g. ~20%). This data suggest the generous indication for postoperative IMRT in initial situations with estimated RR exceeding ~10–15%.

n treated R: number of treated recurrences (idealised value, as not all rHNC patients can undergo salvage treatment with curative intention [6,8,29]).

## Conclusion

A low DSS rate of only 50% at 2 years following primary or postoperative salvage IMRT has been assessed. Based on the calculated NNT, we recommend initial postoperative irradiation +/- chemotherapy, if the estimated risk of local-regional recurrence after initial surgery alone exceeds ~10%.

## Abbreviations

rHNC: Recurrent head neck cancer; IMRT: intensity modulated radiation therapy; rGTV: recurrence gross tumor volume; DSS: disease specific survival; DFS: disease free survival; CT: computed tomography; OCC: oral cavity cancer; NNT: numbers needed to treat.

## Competing interests

The authors declare that they have no competing interests.

## Authors' contributions

GS and CG drafted the manuscript/design of the study, performed the statistical analysis; GC, KWG and GS read and approved the final manuscript. GS and CG are responsible for the clinical IMRT program at the Department of Radiation Oncology, University Hospital Zurich.
